# The short-term effects and burden of particle air pollution on hospitalization for coronary heart disease: a time-stratified case-crossover study in Sichuan, China

**DOI:** 10.1186/s12940-022-00832-4

**Published:** 2022-01-19

**Authors:** Wanyanhan Jiang, Han Chen, Jiaqiang Liao, Xi Yang, Biao Yang, Yuqin Zhang, Xiaoqi Pan, Lulu Lian, Lian Yang

**Affiliations:** 1 School of Public Health, Chengdu University of Traditional Chinese Medicine, Chengdu, 610075 Sichuan China; 2 State Key Laboratory of Grassland and Agro-ecosystem, School of Life Sciences, Lanzhou University, Lanzhou, 730000 Gansu China; 3 West China School of Public Health, Sichuan University, No. 17 People’s South Road, Wuhou District, Chengdu, 610041 Sichuan China; 4 Collaborative Innovation Center for Western Ecological Safety, Lanzhou University, Lanzhou, 730000 Gansu China

**Keywords:** Attributable risk, Economic cost, Hospital admissions, CHD, PM pollution

## Abstract

**Background:**

Coronary heart disease (CHD), the leading cause of death globally, might be developed or exacerbated by air pollution, resulting high burden to patients. To date, limited studies have estimated the relations between short-term exposure to air pollution and CHD disease burden in China, with inconsistent results. Hence, we aimed to estimate the short-term impact and burden of ambient PM pollutants on hospitalizations of CHD and specific CHD.

**Methods:**

PM_10_ and PM_2.5_ were measured at 82 monitoring stations in 9 cities in Sichuan Province, China during 2017-2018. Based on the time-stratified case-crossover design, the effects of short-term exposure to particle matter (PM) pollution on coronary heart disease (CHD) hospital admissions were estimated. Meanwhile, the linked burden of CHD owing to ambient PM pollution were estimated.

**Results:**

A total of 104,779 CHD records were derived from 153 hospitals from these 9 cities. There were significant effects of PM pollution on hospital admissions (HAs) for CHD and specific CHD in Sichuan Province. A 10 μg/m^3^ increase of PM_10_ and PM_2.5_ was linked with a 0.46% (95% CI: 0.08, 0.84%), and 0.57% (95% CI: 0.05, 1.09%) increments in HAs for CHD at lag7, respectively. The health effects of air pollutants were comparable modified by age, season and gender, showing old (≥ 65 years) and in cold season being more vulnerable to the effects of ambient air pollution, while gender-specific effects is positive but not conclusive. Involving the WHO’s air quality guidelines as the reference, 1784 and 2847 total cases of HAs for CHD could be attributable to PM_10_ and PM_2.5_, separately. The total medical cost that could be attributable to exceeding PM_10_ and PM_2.5_ were 42.04 and 67.25 million CNY from 2017 to 2018, respectively.

**Conclusions:**

This study suggested that the short-term exposure to air pollutants were associated with increased HAs for CHD in Sichuan Province, which could be implications for local environment improvement and policy reference.

**Supplementary Information:**

The online version contains supplementary material available at 10.1186/s12940-022-00832-4.

## Introduction

Coronary heart disease (CHD), also named ischemic heart disease, is the leading cause of death globally [1]. According to the American Heart Association, the cases of CHD would increase nearly 100% by 2030 [2]. The prevalence of CHD is second leading cause of death worldwide, resulting in escalating death for decades like in China, in which the average annual growth rate being 9.85% from 1980 to 2016 [3, 4].

To date, diverse and growing data have discerned that growing risks and burden of CHD are linked with short-term exposure to particle matter (PM) with aerodynamic diameter ≤ 2.5 (PM_2.5_) μm or ≤ 10 μm (PM_10_) [5–9]. PM_2.5_ pollution would cause around the 2.0% loss of China’s gross domestic product (GDP) by 2030 without necessary measure being taken [10]. For instance, a study revealed that PM_2.5_ accounted for 26.8% CHD deaths in China [11]. The Global Burden of Disease study identified that ambient PM pollution induced around 11.1% of all deaths in China [12]. A study conducted in Chengdu estimated that a 10 μg/m^3^ increase in PM_2.5_ was involved with a 1.2% (95% CI: 0.3, 2.2%) increase in hospital admissions (HAs) for CHD [9]. Primarily, the relationships of HAs for CHD were involved [13, 14], only limited studies associated with specific CHD, like chronic coronary heart disease (CCHD) [15], acute myocardial infarction (AMI) [16, 17], and unstable angina (UA) [15], which could also link with large quantity of HAs regarding the exposure to PM pollution.

Interestingly, from these epidemiology studies, various primary questions were still remained, since these studies mainly confined in limited regions in developed countries. As exposure to PM pollution being ubiquitous, especially for residents in developing countries exposing to high levels of contaminants, PM pollution contributes comparably to public health crisis. Particularly, like the areas of China which with high PM pollution levels, this would contribute to increase the considerable health and economic burden [18, 19]. Like Sichuan Province, it ranked as the fourth of heavily air polluted regions in China, with high annual mean PM levels [20]. The mean levels of PM_2.5_ and PM_10_ levels reached up to 77.4 and 106.4 μg/m^3^ during January 2015 and February 2017 in Sichuan, resulting in about fifteen and seven times higher than the annual guidelines (5 μg/m^3^ for PM_2.5_ and 15 μg/m^3^ for PM_10_) proposed by World Health Organization [21]. Owing to the high population densities and rapid development of industrialization, Sichuan Province is characterized by the intensive consumption of energy and anthropogenic emissions, resulting in severely regional pollution. Besides, the rank of death cause for CHD is increasing during the last decade, which had become the third death cause in Sichuan Province and brought heavy economic burden to their family [10]. Nevertheless, available studies evaluated the relationship of PM pollution and CHD only regarding to Chengdu, which is the provincial capital city of Sichuan Province [9]. While no studies systematically revealed the short-term effect of PM levels on HAs for CHD and economic burden, in addition to the specific CHD involved in Sichuan Province, which is gaining comparable public attention for serious PM pollution and special topography locating in the Sichuan Basin [22].

To fill this data gap, this study involved a time-stratified case-crossover analysis to estimate the impact of ambient PM pollutants on hospitalizations of CHD and specific CHD in 9 cities of Sichuan Basin between January 1, 2017 and December 31, 2018. Besides, the linked burden of CHD owing to ambient PM pollution were estimated.

## Materials and methods

### Study area

**Fig. 1:**
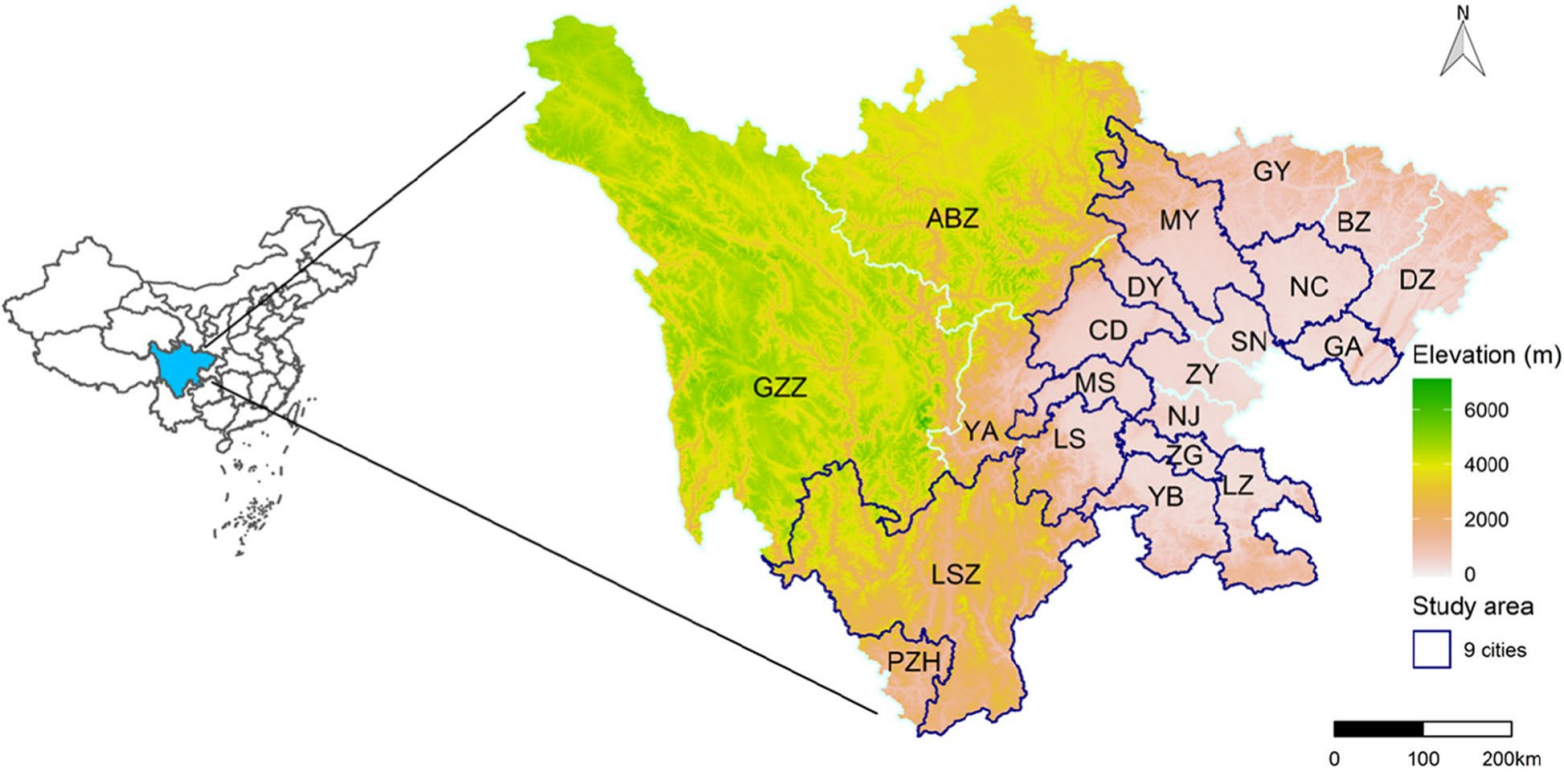
Geographical distribution of study areas, 9 cities of Sichuan Province

This study was implemented in the urban regions of 9 cities in Sichuan Province (Fig. 1). Nine cities, Chengdu (CD), Mianyang (MY), Nanchong (NC), Guangan (GA), Meishan (MS), Zigong (ZG), Liangshanzhou (LSZ), Yibin (YB), and Luzhou (LZ) are involved to discern the effects of air pollution on CHD hospitalizations in these cities. Owing to the lower data quality or limit availability of health data, some cities are exclusive, such as Ganzizhou (GZZ), Abazhou (ABZ) and Guangyuan (GY).

### Data

#### Hospitalization data

The records of CHD were collected from hospital electronic health records (EHRs) of 153 hospitals in 9 cities of Sichuan Province, which contains information on gender, age, date of hospitalization, detailed residence of the patient, primary diagnoses, hospitalization cost, and disease code according to the International Classification of Disease, 10 the Revision (ICD-10). The Daily hospitalization data of CHD (ICD-10: I20-I25) during the period of January 1, 2017 and December 31, 2018 were involved. Besides, three specific subtypes of CHD were involved, including CCHD (ICD-10: I25) [23], AMI (ICD-10: I21-I22) [17, 24], and UA (ICD-10: I20.0) [25], which were all involving comparable quantity of patients, leading cause of mortality, and available evidence on the relations between them and air pollution [24, 26, 27]. The category of these three subtypes was based on the first diagnose as the central factor of hospitalization.

#### Environmental data

The daily mean nitrogen dioxide (NO_2_), sulfur dioxide (SO_2_), ozone (O_3_), PM_10_ and PM_2.5_ were obtained from Sichuan Environmental Monitoring Station between January 1, 2017 and December 31, 2018. There were 82 air monitoring stations in the 9 cities: 19 in CD, 9 MY, 9 in NC, 6 in GA, 6 in MS, 6 in ZG, 17 LSZ and 10 YB. Meteorological data of daily average temperature and relative humidity were available from Sichuan Meteorological Bureau. To assess air pollution exposure, inverse distance weighting (IDW) method were involved [28]. More specifically, the locations of all CHD cases and monitoring stations were geocode using AutoNavi Maps API (https://lbs.amap.com/). Then, for each hospitalization of CHD and measuring station, the exposure of air pollutant on the hospitalized day (lag 0) was estimated by the inverse distance (1/*d*^2^) weighted average of concentrations at all monitoring stations. The single-day lag exposures (lag 1 to lag 7), moving average day exposure (lag 0-1 to lag 0-7) were confirmed. Moreover, lag 0-1, lag 2-7, lag 0-7 were adopted as time window to estimate immediate, delayed or prolonged effects [17]. To deduce liable data, the validation of exposure for each air pollution was involved [28]. The predicted and measured daily levels of air pollutants at all measuring stations were adopted to estimate statistical indicators, like the mean absolute error (MAE). More details could be found at S1 of Supporting Information (SI).

### Modeling

#### Statistical methods

Based on the time-stratified case-crossover design, the exposure to air pollution and daily CHD hospitalizations were estimated. Time-series analysis are associated to observe the relationship between PM pollution and HAs for CHD [29–32]. Case-crossover study as a self-matched case-control proposed by Maclure [33], it would compare the exposure in the case period and cases with exposure in nearby referent periods as reference, to confirm the differences of exposure which might influence the daily count of records [34]. The reference days were selected from the same day of the week in the same month of the same year, when HAs of CHD were recorded, to adjust the influence of long-term tendency, seasonality effect, and day of week from the design framework [35]. Daily cases of CHD approximately followed poisson distribution [36]. A linear model of conditional poisson regression with time-stratified case-crossover design was involved to estimate the short-term exposure of air pollutants and daily CHD hospitalizations in 9 cities of Sichuan Province in China, applying temperature, relative humidity and holidays (assigned a value of 1 on national holidays and 0 on the other days) as potential confounders. Relied on the per 1 μg/m^3^ increase of air levels, the Relative Risk (RR) and 95% confidence intervals (CIs) were estimated. In the exploratory analysis, the natural cubic splines with three degrees of freedom for temperature, relative humidity and holidays was introducing to new models, to examine the nonlinear effect. Then the linear model was better than nonlinear model, with smaller values of the Akaike’s Information Criterion.

Relative risk increase (RRI) was estimated by RR-1. The RRI in HAs for CHD per 10 μg/m^3^ increase of PM levels were calculated as follows:

1 $$RRI\%=\exp \left(\beta \ast 10\right)-1\ast 100\%$$

where β is the exposure-response coefficient of PM-HAs association from conditional poisson regression combined under the time-stratified case-crossover design, which refers to a unit increase in PM pollutants [37].

Single-pollutant models were implemented to discern the effects of air pollution. As aforementioned, to analyze the temporal effect of air levels with different lag structures, from lag 0 to lag 7, from lag 0-1 to lag 0-7, and lag 2-7 were involved. Single day lag refers to the pollution levels on the current day, while cumulative day lag corresponds to moving average of pollution concentrations for the current and previous days. The cumulative day lag influence was unconstrained distributed lag model, which could present unbiased results from the estimation of the overall effect [38].

When estimating the effects of some potential effect modifiers, stratified time-stratified analyses have been involved linking with various subgroups by age group (< 45 years, 45-64 years and ≥ 65 years), gender (male and female) and season (warm season: April-September, and cold season: October-March), applying the above analyses for these subgroups. The statistic differences from stratified analyses (e.g., the difference between male and female) were estimated by *Z*-test [39].

All analysis were conducted using R version 4.0.4 with *gnm* package for conditional poisson regression combined under the time-stratified case-crossover design. All statistical tests with *p* values of < 0.05 were considered as statistically significant. The availability of geocode were obtained from AutoNavi via *amapGeocode* package.

#### Calculating the number of HAs for CHD due to PM pollution

Based on the coefficients from conditional poisson regression combined under the time-stratified case-crossover design, the attributable number of HAs for CHD was calculated. WHO’s air quality guidelines (24 h mean: 45 for μg/m^3^ PM_10_ and 15 μg/m^3^ for PM_2.5_) [21] were involved as reference levels. The equation was shown as follows:

2 $$ANi=\left(\exp \left(\beta \ast \left({x}_i-{x}_0\right)\right)-1\right)/\mathit{\exp}\left(\beta \ast \left({x}_i-{x}_0\right)\right)\ast Ni$$

where *ANi* is the number of HAs which could be attributable to exceeding PM exposures on day *i*; *x*_*i*_ (μg/m) is the exposure level of PM pollution on day *i*; *x*_*0*_ is reference concentration from air quality guideline of WHO; *Ni* is the number of HAs on day *i*; *AN* is the sum of overall *ANi* during the study period. *x*_*0*_, as the base case from the air quality guideline of WHO, is the theoretical minimum threshold levels, below which PM pollution has no effect on HAs [39]. The largest effect in the single pollutant models is involved to estimate the attributable to exceeding PM pollution [19].

#### Evaluating the corresponding hospitalization economic cost

The economic cost of HAs for CHD due to PM exposure were estimated, which involved with total hospital admission expenses and out-of-pocket cost. The equations were shown as follows:

3 $${AC}_{ytotal}={AN}_y\ast {Cost}_{ytotal}\ast {CPI}_y$$

4 $${AC}_{ypocket}={AN}_y\ast {Cost}_{ypocket}\ast {CPI}_y$$

where *AC*_*ytotal*_ and *AC*_*ypocket*_ indicate the total hospital admission cost and out-of-pocket cost which could be attributable to exceeding PM exposure in year *y*; *AN*_*y*_ is the sum of overall *AN*_*y*_ during year *y*; *Cost*_*ytotal*_ and *Cost*_*ypocket*_ represent the case-average total hospital admission expenses and out-of-pocket cost in year *y*; *CPI*_*y*_ is the product of customer price indexs from year *y* + 1 to 2018; *AC*_*total*_ is the sum of *AC*_*ytotal*_, and *AC*_*ypocket*_ is the sum of *AC*_*ypocket*_ during the study period.

### Sensitivity analysis

The two-pollutant models were adopted to estimate the effect after adjusting for co-pollutants. To confirm the robustness of association results, the sensitivity analysis was estimated by evaluating the cases within circular areas of 50 km surrounding air monitoring stations, which could deduce the potential effect of distance between the air pollution monitory site and the address of patient [28].

## Results

### Data description

The descriptive results were displayed in Tables 1 and 2, with data from 153 hospitals located in the 9 cities of Sichuan Province. Between 2017 and 2018 (Table 1), a total of 104,779 CHD hospital admissions (55,891 males and 48,888 females) were recorded, including 83,471 HAs for CCHD, 12817 HAs for AMI, and 3946 HAs for UA. Population age < 45, 45-64, and age ≥ 65 accounted for 4, 24.4 and 71.6% of the total CHD hospitalizations, separately. The mean daily HAs for CHD were slightly higher in cold season than warm season. The overall medical expensed were 1997.6 million China Yuan (CNY), and 781.3 million CNY were self-paid, while the case-average total medical expenses and the out-of-pocket cost were 1.4 and 0.6 thousand CNY, respectively. Daily average concentrations of NO_2_, O_3_, SO_2_, PM_10_, and PM_2.5_ were 30.0 μg/m^3^, 81.8 μg/m^3^, 12.5 μg/m^3^, 71.7 μg/m^3^, and 46.0 μg/m^3^, while the temperature and relative humidity were 17.4 °C and 77.2%, respectively. Detailed summary statistics of specific characteristics for each city can be found in Tables S1, S2 and S3. Person correlation coefficients among these variables was displayed in Fig. S2 of SI.

**Table 1:** Description of the study population

Study population	Number	%
CHD	104,779	100
CCHD	83,471	79.7
AMI	12,817	12.2
UA	3946	3.8
Age		
< 45	4216	4.0
45-64	25,586	24.4
≥ 65	74,977	71.6
Sex (n)		
Males	55,891	53.3
Females	48,888	46.7
Season		
Warm season	49,919	47.6
Cold season	54,860	52.4

**Table 2:** Summary statistics for air pollution levels and weather conditions in the 9 cities of Sichuan Province, 2017-2018

Pollutant	Mean ± SD	Min	Max	Percentiles		
				*P*_25_	*P*_50_	*P*_75_
Air pollution levels (μg/m^3^)						
PM_10_	71.7 ± 46.1	2.4	441.5	37.8	58.8	94.5
PM_2.5_	46.0 ± 33.4	2.1	285.2	22.5	36.2	59.8
NO_2_	30.0 ± 14.3	1.0	127.1	19.6	27.3	37.3
O_3_	81.8 ± 41.6	1.7	310.1	51.1	74.3	107.5
SO_2_	12.5 ± 6.8	1.0	149.6	8.2	11.0	15.0
Meteorological measures						
Temperature (°C)	17.4 ± 7.3	0.3	34.8	10.8	17.3	23.5
Relative humidity (%)	77.2 ± 12.1	13.5	99.9	69.3	78.6	86.5

^a^Unit: case-average expenses, thousand CNY

### Health effects of PM exposure in overall and subgroup population

**Fig. 2:**
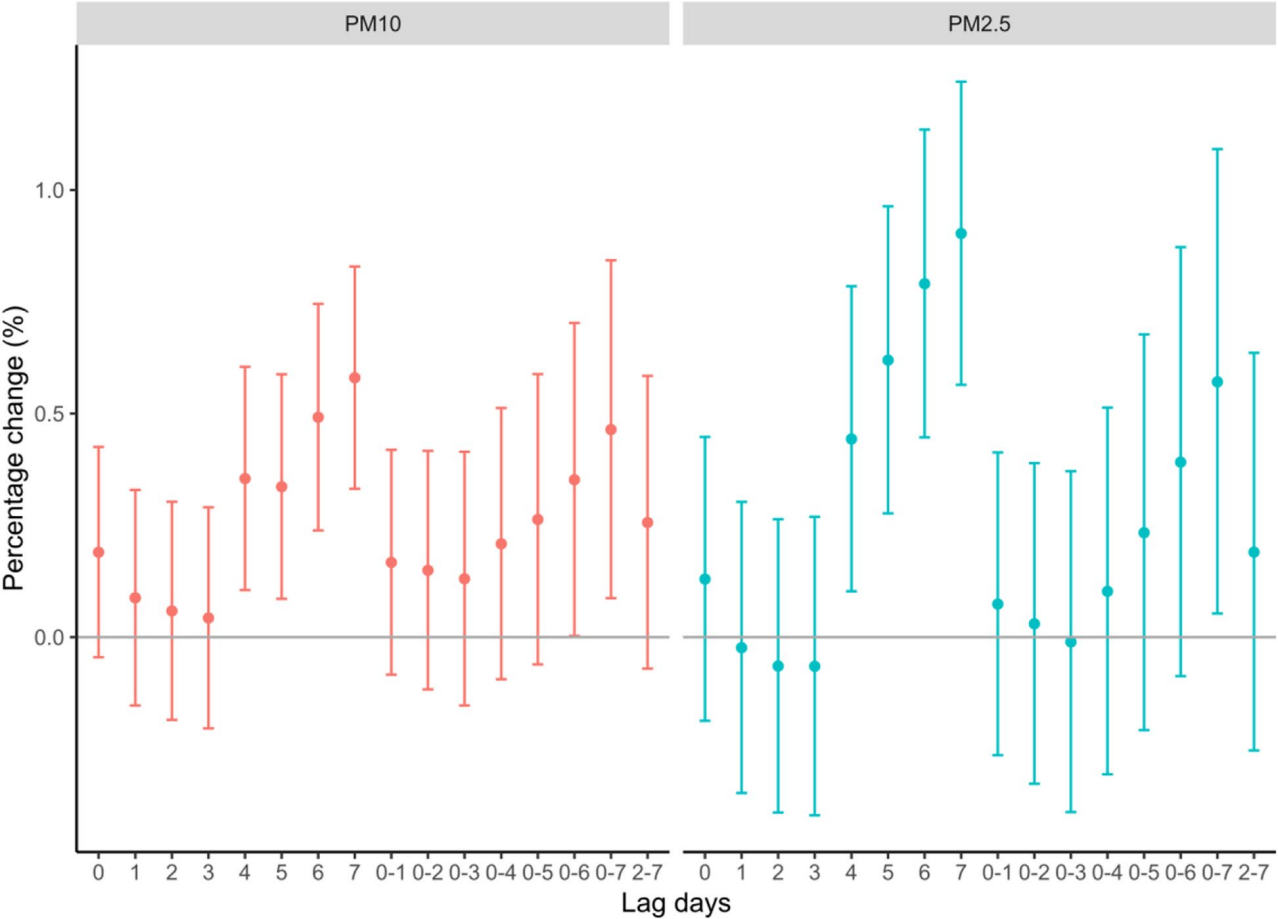
Percentage (95% CI) in HAs for CHD associated with an increase of 10 μg/m^3^ of PM_10_, and PM_2.5_ on single-day lags (from current day to 7 days before: lag 0-lag 7) and multi-day moving average lags (from lag 0-1 to lag 0-7 and lag 2-7)

The associations between PM pollutants and HAs in CHD at various lag days adopting single-pollutant models were displayed in Fig. 2. Overall, there were obvious associations of PM pollutants with HAs for CHD, and general significance at lag 4, lag 6 and lag 7 with the largest association at lag 7. A 10 μg/m^3^ increase of PM_10_ and PM_2.5_ corresponded to a RRI of 0.46% (95% CI: 0.08, 0.84%), and 0.57% (95% CI: 0.05, 1.09%) increases in HAs for CHD at lag 7, respectively.

**Fig. 3:**
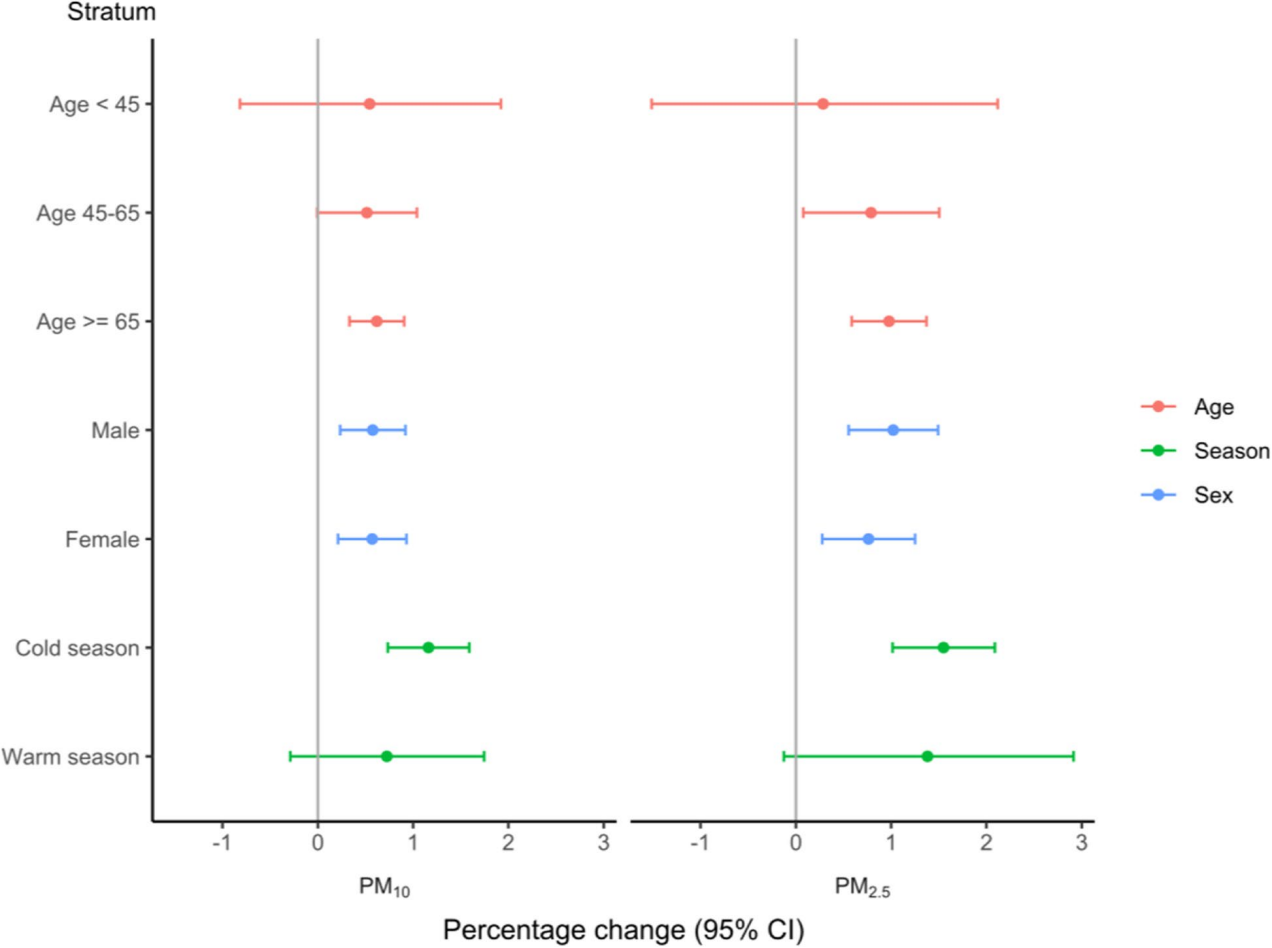
The Percentage change (95% CI) of age, season, and sex group in overall HAs for CHD linking with a 10 μg/m^3^ increase in PM_10_, and PM_2.5_ on lag 7 in Sichuan Province in 2017-2018

To figure the influence of PM_10_, and PM_2.5_ levels on CHD inpatient visits for various subgroups, the stratified analyses involving age, season, and sex lag 7 were illustrated in Fig. 3, after adjustment for temperature, relative humidity and holidays. Owing to the largest effects in single-pollutant model on lag 7, the stratified analyses on lag 7 were performed. In age-specific analysis, the old were more susceptible to PM pollution, with an increase of 10 μg/m^3^ for PM_10_, and PM_2.5_ linking with a RRI of 0.62% (95% CI: 0.33, 0.91%), and 0.98% (95% CI: 0.59, 1.37%) of HAs for CHD, respectively. In gender-specific analysis, the impacts of PM pollution on HAs for CHD were positive and significant. Noteworthily, the difference between male and female for PM_10_ was statistically significant (*p* = 0.02), but the difference between male and female for PM_2.5_ did not reach statistical significance (*p* > 0.05). As for the differences of season groups, the exposure to PM_10_ and PM_2.5_ in cold season was higher, associated with a RRI of 1.16% (95% CI: 0.73, 1.59%) and 1.55% (95% CI: 1.02, 2.09%) in HAs for CHD, based on the increase of 10 μg/m^3^ of PM_10_ and PM_2.5_, separately. However, the differences of stratified season analyses for PM pollution were not statistically significant (*p* > 0.05).

**Fig. 4:**
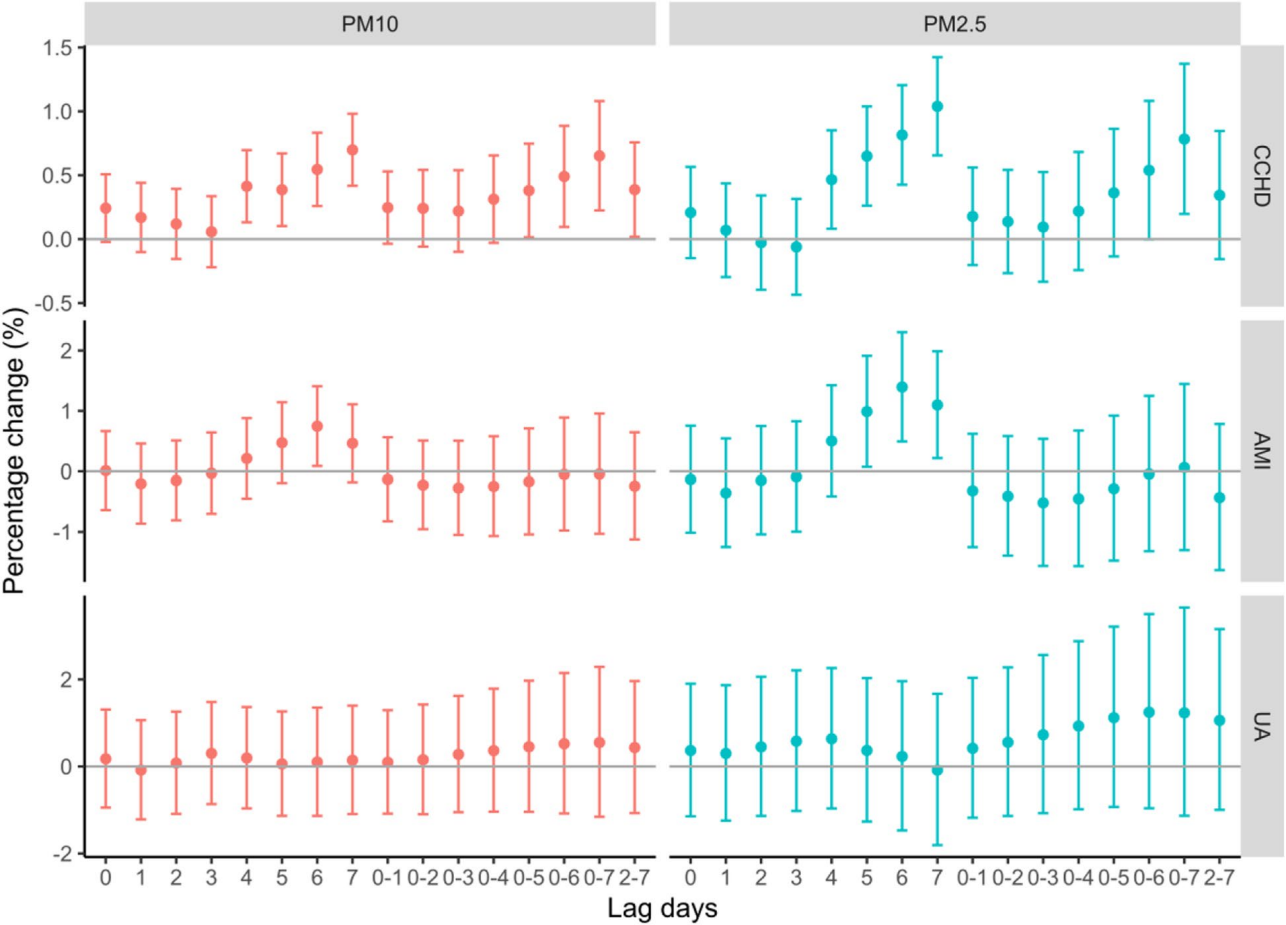
The Percentage change (95% CI) of three specific CHD associated with an increase of 10 μg/m^3^ of PM_10_, and PM_2.5_ on single-day lags (from current day to 7 days before: lag 0-lag 7) and multi-day moving average lags (from lag 0-1 to lag 0-7 and lag 2-7)

The effects of PM exposure on HAs for specific CHD were displayed in Fig. 4. For CCHD, the significant effects of PM_10_ were found at lag 4-7, lag 0-5 to lag 0-7, and lag 2-7 days, while at lag4-7 for PM_2.5_. As for AMI, the effects of PM_10_ were significant at lag 6, and at 5-7 for PM_2.5_. However, for UA, the PM pollution displayed no adverse effect.

### Sensitivity analysis

A two-pollutant model was involved to discern the coupled effects of various pollutants on the number of CHD inpatients (Table S4), excluding the associated effects between PM_2.5_ and PM_10_ owing to the high correlation (*r* = 0.95). In the co-pollutant models, the increases of 10 μg/m^3^ for PM_10_ and PM_2.5_ at lag 7 were linked with RRI of 1.06% (95% CI: 0.59, 1.55%) and 1.63% (95% CI: 0.92, 2.35%) increments in CHD hospitalizations after the adjustment for NO_2_, respectively. After adjustment for SO_2_, the effects of PM pollution on CHD were still significant and robust. Percentage (95% CI) in HAs for CHD associated with an increase of 10 μg/m^3^ of PM_10_, and PM_2.5_ at different lag days within the circular areas of 50 km surrounding air monitoring stations were shown in Fig. S2. When excluding the cases living beyond 50 km from any monitoring station, it suggested that the main models were generally robust.

### Attributable health risks and economic costs due to PM pollution

Table 3 listed the attributable number of HAs and the related economic cost due to exceeding PM exposure involving WHO air quality standard in Sichuan Province, 2017-2018. Based on the reference concentrations, 1784 and 2847 total cases of HAs for CHD could be attributable to PM_10_ and PM_2.5_, separately. As for the total medical expenses and out-of-pocket cost, 42.04 and 16.42 million CNY, and 67.25 and 26.27 million CNY HAs for CHD could be attributable to exceeding PM_10_ and PM_2.5_ in 2017 and 2018, respectively.

**Table 3:** The attributable number of HAs and corresponding economic cost due to exceeding PM exposure using WHO air quality standard in Sichuan Province, 2017-2018

Year	PM_10_			PM_2.5_		
	AN_y_	AC_ytotal_^a^	AC_ypocket_^a^	AN_y_	AC_ytotal_^a^	AC_ypocket_^a^
2017	682	16.68	6.70	1194	29.22	11.70
2018	1102	25.36	9.72	1653	38.03	14.57
Total	1784	42.04	16.42	2847	67.25	26.27

AN_y_, AC_ytotal_, AC_ypocket_ were calculated based on the largest effect estimates in single pollutant models (lag 7)

^a^Unit: million CNY. The annual economic costs were measured at the CPI from 2018

## Discussion

In this study, based on the case-crossover design, the significant associations between short-term exposure to ambient PM pollution and HAs for CHD in 9 cities in Sichuan Province during 2017-2018 had been discerned. As aforementioned, PM pollution was linked with increased daily HAs for CHD, when controlling the influences from confounders which including daily temperature, relative humidity and holidays. Stratified analysis discerned that the old patients and in cold period might more easily engage in the effects of outdoor air pollutants, being consistent with prior studies [31, 40, 41]. As previous studies found, a significant effect of PM pollution was found and varied greatly with various subtypes of CHD [42]. Furthermore, involving the WHO’s air quality as the reference, PM pollution entailed comparable economic burden to HAs for CHD, which was 132.33 million Yuan in total during the study period, 2017-2018.

Increasing studies reported the positive associations between PM pollution and HAs for CHD, since U.S. Environmental Protection Agency (EPA) suggested to discern the short-term relationship between PM pollution and cardiovascular [43]. These epidemiology studies investigated the effect of PM pollution on HAs for CHD, which is in line with the results of this study. A study in Shanghai reported that a 10 μg/m^3^ increase in PM_10_ and PM_2.5_ was coincided with an increase in HAs for CHD by 0.25% (95% CI: 0.10, 0.39%) and 0.57% (95% CI:0.46, 0.68%), separately [13]. A study conducted in Chengdu estimated that a 10 μg/m^3^ increase in PM_2.5_ was involved with a 1.2% (95% CI: 0.3, 2.2%) increase in HAs for CHD [9]. The results presented the effects of PM pollutions on HAs for CHD on different regional scale, contributing to explaining the PM effects all over the world. Besides, this study in Sichuan Province improved the understand of adverse effects of PM pollutants on HAs for CHD.

The mechanism of induced CHD, including specific CHD, from PM pollution is plausible. The increasing PM levels may trigger myocardial infarction, owing to the relationship between hemodynamic and hemostatic alterations with PM pollutions [44], which could be involved with increased plasma viscosity [45], acceleration of heart rates [46, 47] and diminished heart rate variability and ventricular fibrillation and increased number of therapeutic interventions in patients with implanted cardioverter-defibrillators [48]. Besides, different size-specific PM pollution would cause discrepant health effects, with various physical properties, chemical constituents and biological activities [49, 50]. Primarily, PM_10_ is generated by road traffic, while PM_2.5_ is from photochemical reactions or combustion. Since the differences of composition, source and deposition in body, PM_10_ and PM_2.5_ would induce different effects on health [51]. Toxicological studies found PM_10_ and PM_2.5_ both could contribute to pulmonary inflammation, cellular toxicity and oxidative stress, resulting in cardiovascular harm, morbidity and even mortality [52–54]. Some studies found that PM_10_ evoked a more extensive interstitial inflammation [55, 56], whereas others reported PM_2.5_ would link with higher cytotoxicity than PM_10_ [57]. Uniformly, different particle size fractions would impact coagulation, autonomic tone, inflammation parameters and systemic cytotoxicity in various ways.

Owing the differences of regions, population, and social factors and so on, there were various lag associations between air levels and exposure, which would differ according the change of lag time [58]. As Haley et al. [59] founded that different lag time would indicate different results, even contrary data, when applying the effect of PM_2.5_ on cardiovascular disease admissions. Buadong et al. [60] reported the lag 1 of PM_10_ would increase most visits of emergency cardiovascular disease. Xu et al. [61] also observed that hospitalizations of CHD showing strong relation with PM_10_ and PM_2.5_ under short-term exposure. As the result of this study, we found a 10 μg/m^3^ increase of PM_10_ would correspond to a 0.46% (95% CI: 0.08, 0.84%), and 0.57% (95% CI: 0.05, 1.09%) increment in CHD admissions at lag 7. Significant and positive associations for PM levels and HAs for CHD, which reached the peak at lag 7 day and produced the largest effect estimates, being considerable indications to prevent or remedy CHD [62].

The significant linear exposure-response relationship between PM_2.5_ and CHD hospital admissions was not found, which was, however, consistent with some previous studies [63, 64]. Also, at present there were reports found the increase CHD hospital admissions linking with short-time elevated levels of inhalable and/or fine particle matters pollution by various time-series and case-crossover studies [65, 66]. As aforementioned, PM_2.5_ concentrations were comparably lower when compared with other pollutants for these 9 cities in Sichuan Province, which might shrink the effect on health exposure.

The increased risks from PM pollutions were not equally distributed within the subgroups. Compared with people aged < 65 years, the old (age ≥ 65 years) were more susceptive to the effects of ambient air pollution. This was consistent with other studies [66, 67], owing to the old are more vulnerable to air pollution. The effect modification by gender of PM pollution on HAs for CHD were positive but not uniform on the statistical significance. Interestingly, results of gender-specific effects from prior studies were not conclusive [68], while reasons possibly were associated with difference in exposure and physiology. According to Exposure Factors Handbook of Chinese Population, the outdoor time of male and female is different, spending 258 min/day and 210 min/day for males and female, respectively [69]. While females are more susceptive to air pollution with frail physique, sensitive air responsiveness, easily deposition in lung and likely infaust socioeconomic status [70, 71]. Cold season was linking with higher effect of air pollution. As previous studies found higher association in cold season [66, 72]. Likely, owing to the association with low temperature and enhanced blood pressure and viscosity during cold season, this link would deduce heart attacks and strokes with growing winter morbidity and mortality [73]. Likewise, age, temperature and period of the year had shown a role as effect modifiers [74]. The potential factors of stratified variations of effects should be further discerned.

Primary prevention may effectively link with the type shifts of coronary heart events, from AMI to less worsening UA and from acute forms (AMI or UA) to CCHD [75]. In terms of these three specific CHD, CCHD associates with prior myocardial infarction, prior coronary revascularization or multi-vessel CHD without revascularization [76]. Previous studies have discerned the adverse effect of PM_2.5_ concentrations on CCHD [15, 27, 77, 78]. Xie et al. [78] reported that the significantly stronger effects of PM_2.5_ for CCHD compared with AMI at lag 3 days, which is accordant with this study. Ban et al. [15] found that significant effects of PM_2.5_ on HAs for CCHD, with an increase of 10 μg/m^3^ associated with 0.53% (95%CI: 0.39, 0.66%) on lag 0-1. The results indicated that the associated between PM pollution and CCHD could be acute and cumulative. Available evidence revealed that the regions with higher levels of PM_2.5_ displayed high rates of CCHD mortality. PM_2.5_, as fine aerosol particles, would penetrate to the gas-exchange regions of lung, then these ultrafine particles could pass through the lungs into blood circulation and harm other organs [79].

AMI, an event of myocardial necrosis caused by unstable ischemic syndrome, a severe subtype of CHD [80]. Abundant evidence supported the association of PM pollution with AMI. A systematic meta-analysis and review collected the effect of PM pollution on AMI [81]. For the patients with confirmed myocardial infarctions in Boston, the elevated levels of PM_2.5_ were linked with increased risks of AMI only within a few hour or a day after PM exposure [82]. The study conducted in Shanghai reported that 2.30% (95%CI: 1.41, 3.18%) increase in daily emergency visits for AMI involved with an 10 μg/m^3^ increase in PM_10_ [42]. A study in Tuscany (central Italy) found that a 1.30% (95%CI: 0.4, 4.1%) increase of HAs for AMI would link with a 10 μg/m^3^ increase in PM_10_ [16]. AMI is a major death cause and adult disability globally, affecting comparable quantity of older adults [83]. While available studies were limited, more related studies should be involved. Based on the data of this study, 63.1% of AMI patients were over 65 years, which would increase the substantial burden to the society with the elderly being growing.

Contrary to existing data, there was no adverse relationship found between PM pollution and HAs for UA. The study in Beijing estimated that significant association between PM_2.5_ and HAs for UA, involving an estimated risk increase of 0.66% (95%CI: 0.58, 0.73%) with each increment of 10 μg/m^3^ PM_2.5_ [15]. A study conducted in Taiyuan found that a μg/m^3^ increase in PM_10_ and PM2.5 would associate with 1.00% (95CI: 0.60-1.30%) and 1.50% (95%CI: 0.90-2.00%), respectively [84]. So far, there were limited investigations concerning the effects of PM pollution on UA, which was urgent for more studies to further discern the potential pattern. Owing to the different distinct study design and modeling strategies, the deviations of this study and these results could be derived. Besides, available studies mainly associated with short-term effects of PM pollution on HAs for UA, and long-term effects should also take into consideration. Further studies with large study range and population are needed to elucidate the heterogeneous results.

The burden of HAs for CHD attributable to PM exposure was estimated, which is central for cost-effective policy-making and CHD prevention. Both AN and AC are useful assessment for corresponding economic burden of exceeding PM exposures, providing more data to discern the potential association between air pollution and health [85, 86]. Wu et al. [86] adopted such method to assess the economic cost of HAs for mental disorders. A study of Wuhan calculated that attributable hospitalizations and economic costs to PM_10_ and PM_2.5_ were 249 and 340, and 4.82 and 6.57 million RMB from 2015 to 2017, respectively [14, 19, 62]. Yu et al. [87] demonstrated that 27.31% of CHD burden were attributable exposure to PM2.5 in Hubei. These studies presented positive association between burden and PM pollutions. Meanwhile, we found attributable number of HAs, total corresponding medical costs, and out-of-pocket cost were 1784 and 2847, 49.88 and 84.37 million CNY, and 16.42 and 26.27 million CNY, respectively, attributed to PM_10_ and PM_2.5_ from 2017 to 2018. More hospitalizations and economic lost could be avoided if recorded PM levels is at lower levels. The results of burden from PM pollution in this study were inconsistent with previous study, PM_2.5_ contributing more to economic burden [14]. The fraction of health and economic burden fell from 2017 to 2018, which elucidated the contribution of efficient measures for controlling PM pollutions by Chinese government since 2015 [88]. To date, this is the first study involving the estimation on burden of HAs for CHD due to PM exposure in Sichuan Province. The primary public health implications of our study might be the straightforward evidence of economic burden were quantified.

In addition, there were several characteristics of this study. First, the data on CHD hospitalizations were collected from the tertiary and secondary hospitals in 9 cities and involved 104,779 cases across the Sichuan Province. The large size of records enabled to elucidate the small adverse coronary effects with high precision, and link with regional variations within the province. Second, to our knowledge, this is the first investigation to confirm the effect of outdoor air pollution to HAs for CHD across the Sichuan Province associating with case-crossover design, which is a credible analysis to evaluate the short-term effects of air pollution on health [89]. Third, IDW interpolation method was used to constructed high spatial resolution for exposure estimation, to improve the spatial accessibility of pollutant levels and facilitate the confirmation of exposure effects. Besides, this study is also the first one to simultaneously estimate and compare the short-term associations of PM pollution on specific subtypes of CHD across Sichuan Province.

There were some limitations of this study. First, this study was associated with 9 urban districts of Sichuan Province, without involving other cities owing to the low data quality and unavailability to the data. Then, monitoring data from limited stations, which would be involved as the agent for personal exposure. This process was linking with ineluctable error of measurement, which was an inherent limitation of epidemiology studies relating to environment and disease [90]. We hope some sophisticated and precise methods, like satellite-based spatiotemporal models [17, 91] could be involved, to deduce various evidence. Third, this study might understate the real economic burden of CHD owing to PM pollutions, since the economic cost only involved the total medical expenses and out-of-pocket cost, without the outpatient expenses and indirect medical cost. Generally, all limitations of study should be mentioned, then further researched.

## Conclusions

The results of this study estimated the associations between air levels and CHD hospitalizations in 9 cities Sichuan Province, China, involving the time-stratified case-crossover analysis. Based on the effects and burden of PM pollution on HAs for CHD and specific CHD, the event rate and burden of CHD would decrease with lower pollution levels, shedding light on the strategies of public health prevention. In summary, our study can supplement limited evidence of PM health effects in China. Further efforts are needed to understand the socioeconomic and demographic factors that might modify the effects of air pollution.

## Supplementary Information

**Additional file 1.**

## Data Availability

The datasets used and analyzed during the current study are available from the corresponding author on reasonable request.
