# Supplementary material for: The short-term effects and burden of particle air pollution on hospitalization for coronary heart disease: a time-stratified case-crossover study in Sichuan, China
Source: Environ Health. 2022 Jan 19;21:19. doi: 10.1186/s12940-022-00832-4 (PMC8767695; doi:10.1186/s12940-022-00832-4)
Supplement: Supplementary file 1 — Additional file 1. [file 12940_2022_832_MOESM1_ESM.docx]

Supporting information for

**The short-term effects and burden of particle air pollution on hospitalization for coronary heart disease: A time-stratified case-crossover study in Sichuan, China**

Wanyanhan Jiang^1¶^, Han Chen^2¶^, Jiaqiang Liao^3^, Xi Yang^1^, Biao Yang^1^, Yuqin Zhang^1^, Xiaoqi Pan^1^, Lulu Lian^4^,Lian Yang^1*^

^1^ School of Public Health, Chengdu University of Traditional Chinese Medicine, Chengdu, 610075, Sichuan, China

^2^ State Key Laboratory of Grassland and Agro-ecosystem, School of Life Sciences, Lanzhou University, Lanzhou, 730000, Gansu, China

^3^ West China School of Public Health, Sichuan University, No. 17 People's South Road, Wuhou District, Chengdu, 610041, Sichuan, China

^4^ Key Laboratory for Environmental Pollution Prediction and Control, Gansu Province, College of Earth and Environmental Sciences, Lanzhou University, Lanzhou, 730000, Gansu, China

* Corresponding author: Lian Yang

E-mail: [yyanglian@163.com](mailto:yyanglian@163.com)

^¶^ These authors contributed equally to this work.

# S1 Assessment of exposure assessment

For each air pollution, the exposure levels of monitoring station on each date were predicted by the inverse distance weighting (IDW). Then the prediction accuracy was assessed by calculating coefficient of determination (R­^2^), mean absolute error (MAE) and bias between predicted and measured concentrations as follows:

$MEA=\frac{1}{N}\sum_{i=1}^{N} |P_{i}-M_{i}|$ (S1)

$bias=\frac{1}{N}\sum_{i=1}^{N} P_{i}-M_{i}$ (S2)

where M is measure concentration (μg/m^3^); $\bar{M}$ is the mean of measured concentrations (μg/m^3^); P is the predicted concentrations (μg/m^3^).

**Table S1.** Performance of IDW method in exposure assessment for the daily air pollutant levels (μg/m^3^).

| Air pollutants | MAE | Bias |
| --- | --- | --- |
| PM_10_ | 47.2 | 1.96 |
| PM_2.5_ | 33.1 | 1.70 |
| NO_2_ | 12.3 | 0.976 |
| O_3_ | 50.4 | -1.20 |
| SO_2_ | 7.0 | 0.87 |

# S2 Data description on city scale

**Table S2.** Summary statistics of daily CHD hospital admissions in 9 cities in Sichuan Province, 2017-2018.

| City | Daily CHD hospital admissions (n) | | | | | |
| --- | --- | --- | --- | --- | --- | --- |
|  | Mean ± SD | Minimum | P_25_ | P_50_ | P_75_ | Maximum |
| Chengdu | 45 ± 15 | 12 | 31 | 47 | 57 | 81 |
| Guang’an | 11 ± 9 | 1 | 2 | 12 | 18 | 84 |
| Liangshanzhou | 5 ± 3 | 1 | 3 | 4 | 7 | 29 |
| Luzhou | 4 ± 2 | 1 | 2 | 3 | 5 | 14 |
| Meishan | 13 ± 5 | 2 | 9 | 12 | 16 | 39 |
| Mianyang | 21 ± 11 | 2 | 13 | 19 | 28 | 60 |
| Nanchong | 4 ± 3 | 1 | 2 | 3 | 5 | 14 |
| Yibin | 28 ± 11 | 7 | 20 | 26 | 33 | 83 |
| Zigong | 15 ± 9 | 1 | 8 | 13 | 22 | 56 |

SD: standard deviation

**Table S3.** Mean ± SD (ug/m^3^) of daily air pollution concentration in 9 cities in Sichuan Province, 2017-2018.

| City |  | Mean ± SD (ug/m^3^) | | | | | |
| --- | --- | --- | --- | --- | --- | --- | --- |
|  | PM_10_ | | PM_2.5_ | NO_2_ | O_3_ | SO_2_ |  |
| Chengdu | 7.5 ± 5.1 | | 4.7 ± 3.6 | 3.7 ± 1.7 | 8.6 ± 4.7 | 1.1 ± 0.5 |  |
| Guang’an | 7.3 ± 4.1 | | 4.4 ± 3.1 | 2.4 ± 1.1 | 7.4 ± 3.9 | 1.2 ± 0.6 |  |
| Liangshanzhou | 3.9 ± 1.9 | | 2.2 ± 1.3 | 1.6 ± 0.7 | 8.2 ± 2.7 | 1.4 ± 0.6 |  |
| Luzhou | 7.2 ± 4.3 | | 4.7 ± 3.2 | 2.9 ± 1.1 | 7.4 ± 4.1 | 1.7 ± 0.8 |  |
| Meishan | 7.4 ± 4.5 | | 4.7 ± 3.2 | 2.9 ± 1.2 | 9 ± 4.3 | 1 ± 0.5 |  |
| Mianyang | 6.9 ± 4.3 | | 4.3 ± 2.9 | 2.5 ± 1.1 | 7.9 ± 3.5 | 0.9 ± 0.5 |  |
| Nanchong | 6.6 ± 3.8 | | 4.1 ± 2.7 | 2.8 ± 1.2 | 7.6 ± 3.4 | 1.2 ± 0.5 |  |
| Yibin | 6.8 ± 4.2 | | 4.7 ± 3.3 | 2.9 ± 1.2 | 7.7 ± 3.8 | 1.8 ± 0.9 |  |
| Zigong | 7.9 ± 4.9 | | 5.4 ± 3.8 | 2.7 ± 1 | 8.3 ± 4.1 | 1.3 ± 0.5 |  |

SD: standard deviation

**Table S4.** Mean ± SD (ug/m^3^) of daily air pollution concentration in 9 cities in Sichuan Province, 2017-2018.

| City | Mean ± SD | |
| --- | --- | --- |
|  | Temperature (℃) | Relative humidity (%) |
| Chengdu | 7.7 ± 1.2 | 1.7 ± 0.7 |
| Guang’an | 7.8 ± 1.2 | 1.7 ± 0.8 |
| Liangshanzhou | 6.3 ± 1.7 | 1.7 ± 0.6 |
| Luzhou | 8 ± 1.1 | 1.8 ± 0.7 |
| Meishan | 7.8 ± 1.1 | 1.8 ± 0.7 |
| Mianyang | 7.4 ± 1.2 | 1.7 ± 0.8 |
| Nanchong | 7.7 ± 1.2 | 1.7 ± 0.8 |
| Yibin | 8 ± 1.1 | 1.8 ± 0.7 |
| Zigong | 7.9 ± 1.1 | 1.8 ± 0.7 |

SD: standard deviation

# S3 Correlation for air pollution and meteorological data


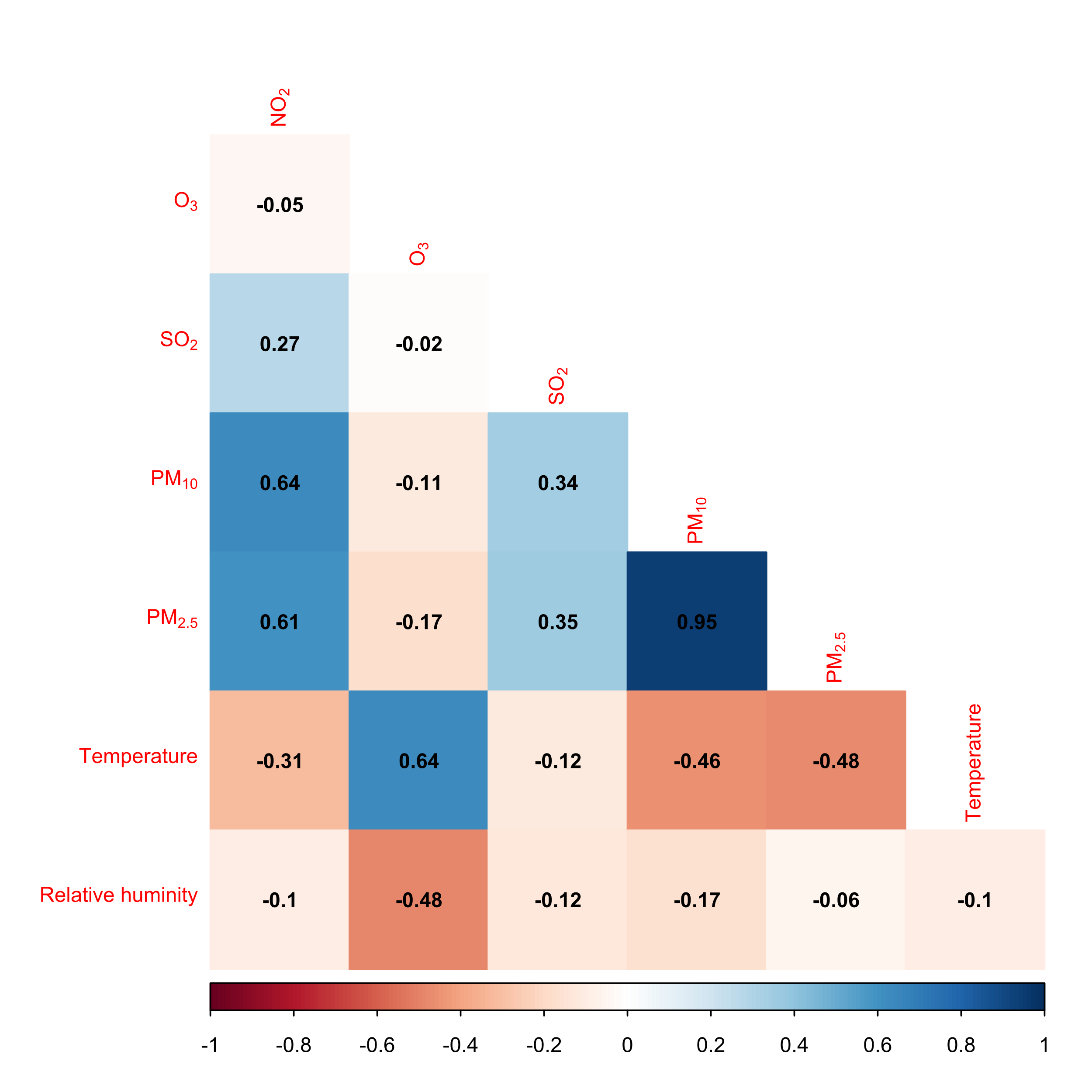


**Figure S1.** Pearson correlation coefficient between air pollutant levels and weather conditions in 9 cities in Sichuan Province, with the color bar on the bottom of the figure.

The Pearson correlation coefficients for the air pollution levels and meteorological conditions were displayed in **Figure S1**. As shown, PM_2.5_ was highly correlated with PM_10_ (*r* = 0.95, *p* < 0.001). There was a certain degree of correlation for other gaseous pollutants except O_3_ (for SO_2_ and NO_2_, *r* = 0.27-0.64, *p* < 0.001), and PM_2.5_ with meteorological factors.

# S4 Sensitivity analysis

In the two-pollutant model, the effects of PM_10_ and PM_2.5_ on HAs for CHD presented significant after adding NO_2_, O_3_, or SO_2_. In accordance with results estimated from the single-pollutant models, the two-pollutant models at lag7 were fitted to assess the stability of the effects of PM, which was with the largest effects at lag 7.

**Table S5.** Percentage change (%) and its 95% CI of HAs for CHD associated with 10 μg/m^3^ increase of PM_2.5_ and PM_10_ at lag7 after adjusting for NO_2_, SO_2_, PM_2.5_ and PM_10_ levels at lag7 in two-pollutant models.

|  | PM_10_ | PM_2.5_ |
| --- | --- | --- |
| none | 0.58 (0.33, 0.83) | 0.90 (0.56, 1.24) |
| +NO_2_ | 1.06 (0.59, 1.55) | 1.63 (0.92, 2.35) |
| +O_3_ | 0.48 (0.10, 0.86) | 0.62 (0.09, 1.15) |
| +SO_2_ | 0.90 (0.44, 1.36) | 1.28 (0.62, 1.94) |


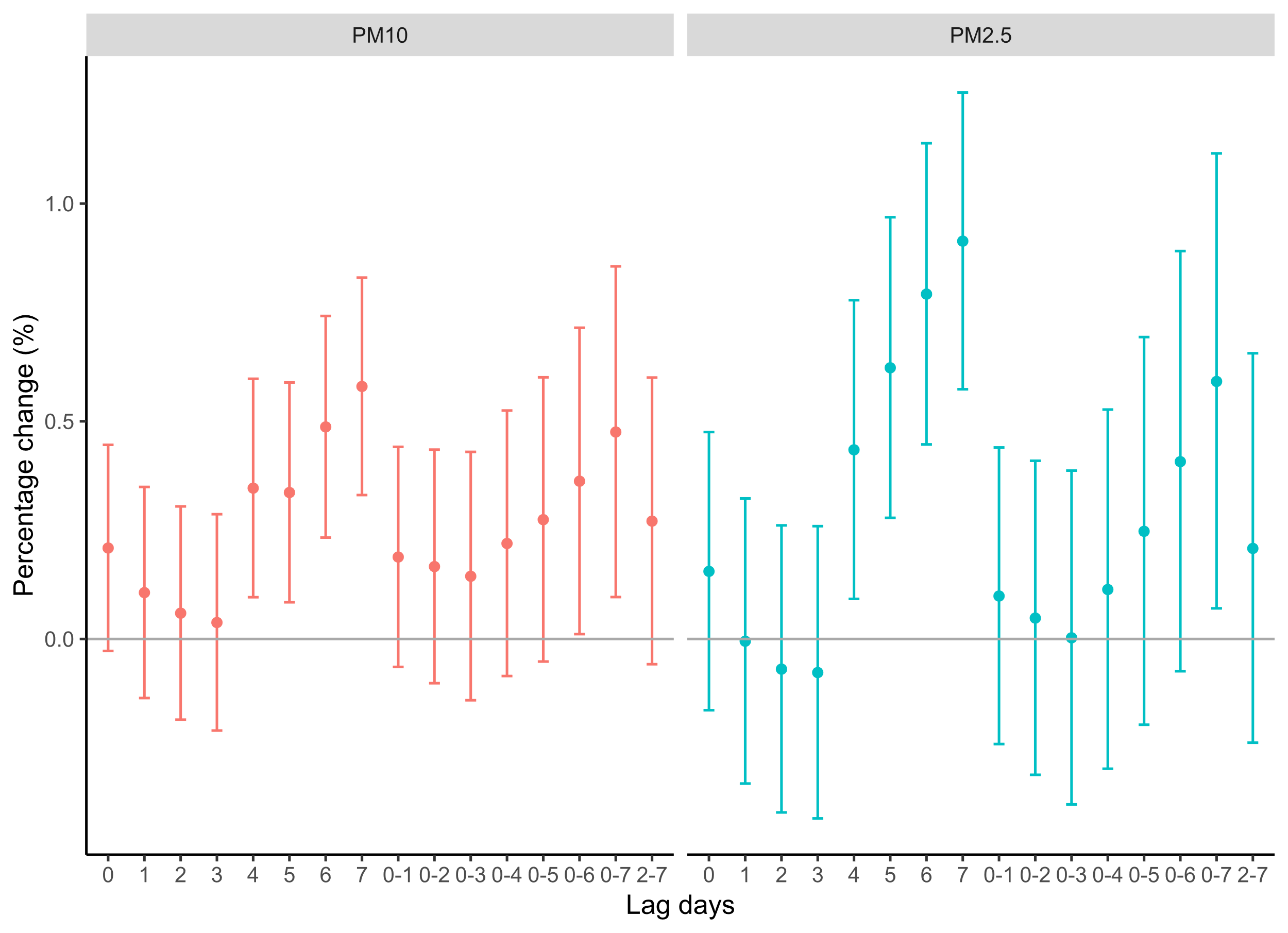


**Figure S2.** Percentage (95% CI) in HAs for CHD associated with an increase of 10 ug/m^3^ of PM_10_, and PM_2.5_ at different lag days using single-pollutant models within the circular areas of 50km surrounding air monitoring stations in Sichuan, 2017-2018
